# A Novel Electroactive Imide Oligomer and Its Application in Anticorrosion Coating

**DOI:** 10.3390/polym12010091

**Published:** 2020-01-04

**Authors:** Bi-Sheng Huang, Guan-Hui Lai, Ta-I Yang, Mei-Hui Tsai, Yi-Chen Chou

**Affiliations:** 1 Ph. D. Program, Graduate Institute of Precision Manufacturing, National Chin-Yi University of Technology, Taichung 41170, Taiwan; unicorn6623917@gmail.com (B.-S.H.); hueilai123@gmail.com (G.-H.L.); 2 Department of Chemical Engineering, Chung-Yuan Christian University, Taoyuan 330, Taiwan; taiyang@cycu.edu.tw; 3 Department of Chemical and Materials Engineering, National Chin-Yi University of Technology, Taichung 41170, Taiwan; 4 Department of Applied Cosmetology, Hungkuang University, Taichung 44302, Taiwan

**Keywords:** electroactive, aniline tetramer, anti-corrosion

## Abstract

A novel aniline tetramer (AT) capped electroactive imide oligomer (EIO) for metal corrosion protection was successfully synthesized in this study. The chemical structure of the EIO was characterized by liquid chromatography-mass spectrometry and Fourier-transform infrared spectroscopy. Furthermore, the redox behavior of EIO was identified using electrochemical cyclic voltammetry studies. An EIO coated on a cold-rolled steel (CRS) electrode was found to possess superior corrosion resistance to polyimide (PI) on a series of electrochemical corrosion measurements in 3.5 wt.% NaCl solution over an extended period (30 days). The mechanism for the advanced corrosion protection of the PI coating on the CRS electrode could be attributed to the redox catalytic capabilities of the AT units present in the EIO. These capabilities may induce the formation of passive metal oxide layers on the CRS electrode. Scanning electron microscopy and X-ray photoelectron spectroscopy were used to analyze the surface condition of the CRS after the corrosion test. EIO- and PI-coated electrodes were identified by a series of electrochemical measurements, including corrosion potential (E_corr_), polarization resistance (R_p_), and corrosion current (I_corr_) measurements, along with electrochemical impedance spectroscopy (EIS).

## 1. Introduction

Metallic corrosion is a serious safety and economic issue for construction and industrial development [1]. Many approaches to corrosion protection have been developed, including organic coatings [2,3,4,5], inorganic coatings [6,7], ceramic coatings [8], and corrosion inhibitors [9,10]. Recently, organic coatings have attracted significant attention due to their potential for simple preparation, low cost, and functional potential, while being considered one of the most efficient strategies for corrosion protection. However, most of the organic coatings are not perfect barriers for providing long-term corrosion protection because such coatings are more or less permeable to corrosive media like water, oxygen, and ions. Therefore, several approaches for corrosion protection have been developed utilizing organic coatings. Some methods involve the addition of chromate as a corrosion inhibitor, which can provide the highest level of corrosion protection. Unfortunately, the adverse health and environmental effects, high toxicity, and carcinogenic risks of chromium limit its safety and therefore use [11,12].

Conducting polymers such as polyaniline (PANI), polypyrrole, and polythiophene are interesting materials for both research and technological applications. There has been increasing interest in the use of these polymers to reduce the corrosion of oxidizable metals [13,14,15]. Among these types of polymers, PANI has been considered one of the most promising electrode materials due to its facile synthesis, environmental stability, unique electronic properties, and simple acid–base doping/dedoping chemistry [16,17]. Although PANI has good anti-corrosion properties [18,19,20], poor solubility of PANI in common organic solvents has limited its practical application in many fields. To find an appropriate counter to this shortcoming, aniline oligomers and their derivatives have been the subject of much recent research [21,22,23,24].

Kaner and his research team have demonstrated that aniline tetramer (AT) is the smallest oligomer unit that can fully represent the structure of PANI [25]. Faul et al. synthesized the AT-alkyl diblock compound which self-assembled into single-crystalline two-dimensional microplates and studied its electrical conductivity [26]. Chao and Wang also prepared electroactive polymers containing AT in the main and side chains, which exhibited electrochemical properties, electrochromic behavior, and fluorescent sensing properties [27,28,29]. Huang et al. incorporated the amino-capped aniline trimer into a polyurethane prepolymer to obtain electroactive polyurethane coatings, exhibiting a stronger corrosion protection on the cold rolled steel in the corrosion medium than the pristine polyurethane coating [30]. Liu et al. prepared self-assembled AT based conducting nanofiber incorporated into a waterborne epoxy as a composite coating to enhance the corrosion resistance of mild steel (Q235 grade), demonstrating a relatively high conductivity and reversible redox behavior [31].

Polyimide (PI) is a high-performance engineering polymer with outstanding properties, including film-forming ability, thermal stability, and mechanical properties compared to conventional polymers. Thus, PI films have been used commercially in various applications such as flexible substrates, gas separation membranes [32], chemical sensors [33,34], and anticorrosion materials [5,35,36].

In this work, we present the electrochemically investigated corrosion protection properties of aniline tetramer-capped electroactive imide oligomer (EIO) coating on cold-rolled steel (CRS) electrodes. The corrosion resistance of the EIO coating was compared to a corresponding PI coating using a series of electrochemical measurements. The electroactivity of the EIO was characterized by cyclic voltammetry. The Tafel curve and EIS spectra were used to investigate the anti-corrosion performance. Scanning electron microscopy and X-ray photoelectron spectroscopy were used to analyze the corrosion products.

## 2. Experimental

### 2.1. Materials and Instrumentation

N-Phenyl-p-phenylenediamine (98%, Alfa-Aesar, haverhill, MA), ferric chloride (99%, ECHO, Taichung), 4-4′-diaminodiphenylether (ODA, 97%, Sigma-Aldrich, Missouri, St. Louis), 4,4′-(4,4′-Isopropylidenediphenoxy)bis(phthalic anhydride) (IDPA, 98%, Sigma-Aldrich, Missouri, St. Louis), N-methyl-2-pyrrolidone (NMP, 99%, TEDIA, Fairfield, OH), hydrochloric acid (35%, SHOWA, Xingtian city, Saitama), ammonium persulfate (APS, 98%, J.T.Baker, Phillipsburg, New Jersey), ammonium hydroxide (25%, SHOWA, Xingtian city, Saitama), N,N-Dimethylacetamide (DMAc, 99%, TEDIA, Fairfield, OH), dimethylformamide (DMF, 99%, TEDIA, Fairfield, OH), dimethyl sulfoxide (DMSO, 95%, TEDIA, Fairfield, OH), tetrahydrofuran (THF, 99.5%, Acros, Geel, Belgium), chloroform (99%, Shimakyu’s Pure Chemicals, Osaka-shi, Osaka), dichloromethane (99.5%, Shimakyu’s Pure Chemicals, Osaka-shi, Osaka), acetone (99%, ECHO, Taichung), ethanol (95%, ECHO, Taichung) were used as received. Attenuated total reflection Fourier transform-infrared spectra were collected using an FTIR spectrometer (JASCO, Hachioji, Tokyo, FT/IR-4600) at room temperature, the resolution used in the measurements and the number of scans was 4 cm^−1^ and 64 scans, respectively. Mass spectra were obtained on a triple quadrupole mass spectrometer (TSQ Quantum) with an ESI source. UV-Vis absorption spectra were collected using a UV-Vis spectrometer (JASCO V-750). The field emission scanning electron microscope (JEOL, Akishima, Tokyo, JSM-7800F, in-lens Schottky field emission electron gun, accelerating voltages was 3.0 KV) and X-ray photoelectron spectrometer (XPS, Thermo K-Alpha, Al K-alpha microfocus monochromated X-ray source) were used to characterize passivation by metal oxide layers. Cyclic voltammetry experiments were performed on CHI 6273E electrochemical analyzer using a conventional three-electrode system. The electrochemical corrosion measurements were performed using an electrochemical workstation zive sp1 (WonATech, Seoul, Korea). The area of work electrode was 1 cm^2^. All tests were performed at 25 °C in corrosive medium (3.5 wt.% sodium chloride solution).

### 2.2. Synthesis of Aniline Tetramer (AT)

The synthesis of AT was performed by a modification of the published literature procedures [37]. The ferric chloride hexahydrate (4.90 g, 0.01 mole) in HCl solution (30 mL, 1.0 M) was rapidly added to a solution of the N-phenyl-1,4-phenylenediamine (aniline dimer, 2.0 g, 0.01 mole) in HCl solution (200 mL, 1.0 M). After vigorous mechanical stirring for 4 h in the ice bath, the product was collected by centrifugation, and washed repeatedly with 1.0 M HCl and deionized water until the supernatant was colorless. The resulting precipitate in the emeraldine salt state was then treated with ammonium hydroxide solution (1.0 M, 200 mL) for 24 h. The precipitate was collected by filtration and dried in a vacuum oven at 50 °C for 24 h to yield aniline tetramer in the emeraldine base state.

### 2.3. Synthesis of Polyimide (PI)

A total of 1.04 g (2 mmol) IDPA was dissolved in 10 mL of NMP under nitrogen at room temperature, followed by slowly added into a stirred solution of 0.4 g (2 mmol) of ODA dissolved in 10 mL of NMP. The reaction mixture was stirred for 6 h to obtain a solution containing poly(amic acid) (PAA). The as-prepared PAA solution was dried under vacuum at 250 °C for 5 h to complete the chemical imidization reaction of the polyimide (PI).

### 2.4. Synthesis of Electroactive Amic Acid Oligomer (EAAO) and Imide Oligomer (EIO)

**Scheme 1:**
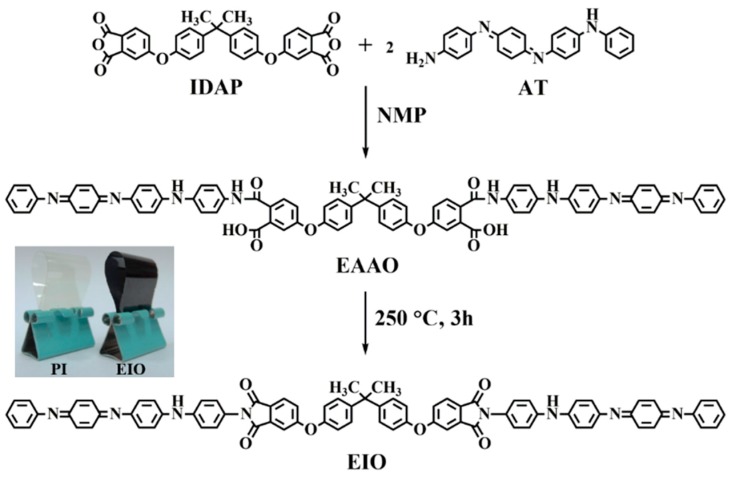
Synthesis route of the EAAO and EIO.

A total of 1.04 g (2 mmol) of IDPA was dissolved in 10 mL of NMP under nitrogen at room temperature, followed by slowly added into a stirred solution of 0.73 g (2 mmol) of AT dissolved in 10 mL of NMP. The solution was magnetically stirred for 4 h to obtain a solution containing aniline tetramer capped electroactive amic acid oligomer (EAAO). The as-prepared EPAAO solution was dried under vacuum at 250 °C for 3 h to complete the chemical imidization reaction of the electroactive imide oligomer (EIO), as shown in Scheme 1.

### 2.5. Reduction of AT and EAAO

The as-prepared EAAO solution was poured into 100 mL of distilled water for precipitation. The product was collected by filtration and washed with an excess of distilled water and dichloromethane several times, and finally dried under dynamic vacuum at room temperature for 24 h. The as-prepared AT and EAAO (0.1 g) were dispersed into a solution of 1 mL hydrazine hydrate in 10 mL 1.0 M ammonium hydroxide, respectively. After stirring for 24 h, the products were filtered and washed with distilled water several times, and dried under dynamic vacuum at 40 °C for 24 h. Finally, the obtained the leucoemeraldine oxidation state of AT and EAAO.

### 2.6. Electrochemical Measurements

All of the electrochemical corrosion measurements were performed by electrochemical workstation zive sp1 (Wonatech, Korea) at 25 °C in corrosive medium (3.5 wt.% sodium chloride solution). The working electrode (cold rolled steel, CRS) was groundby sandpaper 800 and 1000 grit, sequentially. Meanwhile, each of the AT, EAAO and PAA solutions were prepared with NMP and then drop-coating onto the groundCRS. The samples were dried under vacuum at 250 °C for 3 h. The thickness of the coatings was determined using micrometers (Mitutoyo 293-240-30 IP65, range 0-25 mm, resolution 0.001 mm). The average thickness was 0.022 mm ± 0.001 mm. The area of working electrode was 1 cm^2^. All tests were performed in the corrosive medium for one hour to ensure the steady state prior to polarization current measurement. The potential was scanned from -250 to +250 mV at a scan rate of 2.5 mV s^−1^ for polarization current experiments. The corrosion potential (*E_corr_*) and corrosion current density (*I_corr_*) were determined by the Tafel extrapolation method. The *I_corr_* was determined by superimposing a straight line along the linear portion of cathodic or anodic curve and then extrapolating it through *E_corr_*.

The polarization resistance (*R_p_*) value was calculated from the Tafel plots, according to the Stearn-Geary equation [38,39]:

(1) $$R_{p}=\frac{b_{a}b_{c}}{2.303\left(b_{a}+b_{c}\right)I_{corr}}$$

where *b_a_* and *b_c_* are the anodic slope and cathodic slope, respectively. Corrosion rate (*CR*, in mm per year) was calculated using the following equation [40,41]:

(2) $$\mathrm{CR}=\frac{I_{corr}\times M}{D\times V}\times 3270$$

where *I_corr_* is the current density (A cm^−2^), *M* is the molecular mass, *V* is the valence (the number of electrons that lose during the oxidation reaction), and *D* is the density of CRS (g cm^−3^).

## 3. Results and Discussion

### 3.1. Characterization of AT

**Figure 1:**
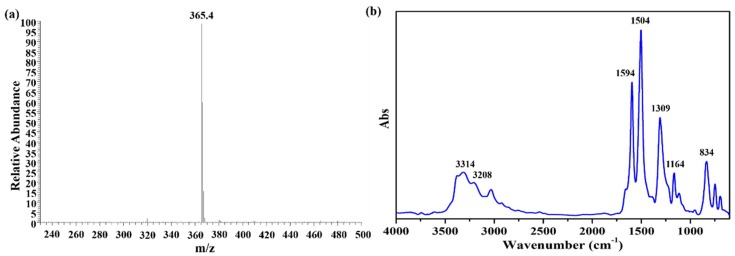
Characterization of aniline tetramer by (**a**) Mass and (**b**) ATR-FTIR spectroscopies.

The Mass and ATR-FTIR spectra of AT as shown in Figure 1. The characterizations of AT were as follows: ion trap-MS m/z: [M + H]^+^ calculated for C_24_H_20_N_4_ = 365.4. Found 365.4. In the ATR-FTIR spectrum of AT, the characteristic bands at 3314 cm^−1^ and 3208 cm^−1^ were attributed to the N-H stretching modes. Moreover, the characteristic bands at 1594 cm^−1^ and 1504 cm^−1^ were attributed to C = C of quinoid rings and benzenoid rings, respectively [37].

### 3.2. Characterization of EAAO and EIO

**Figure 2:**
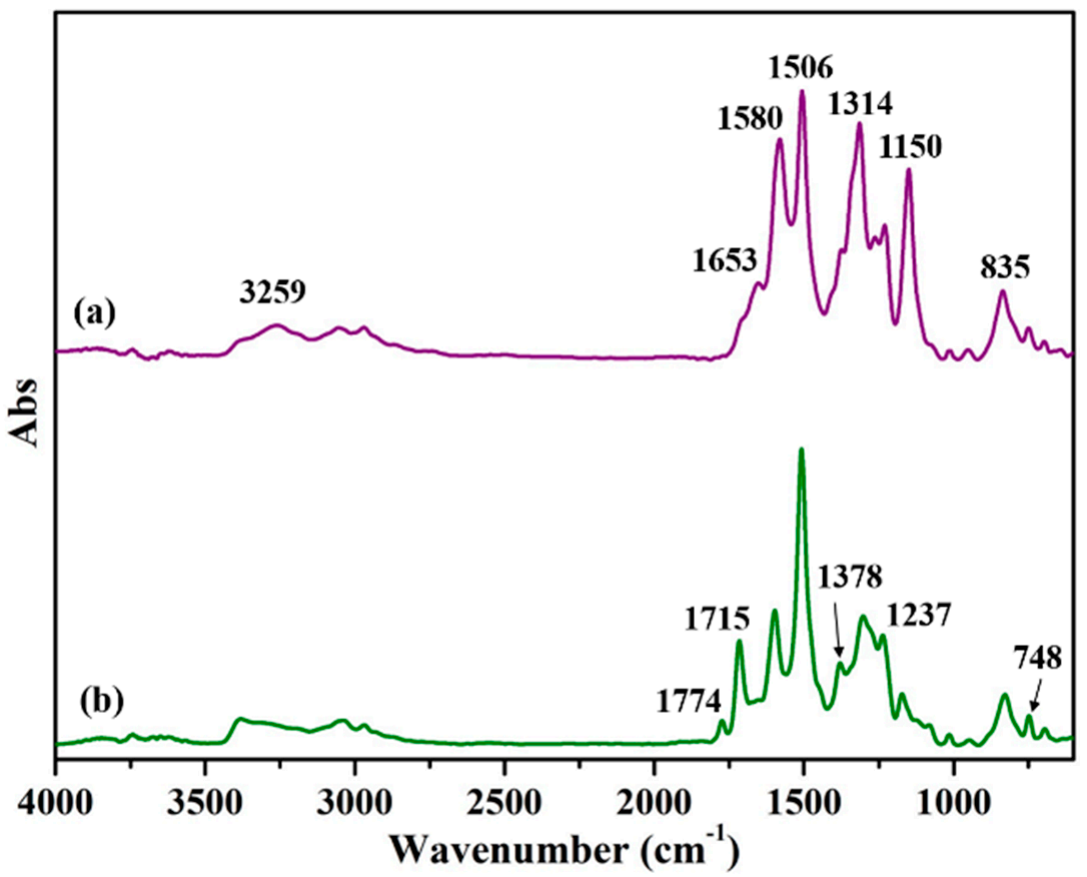
ATR-FTIR spectroscopies of (**a**) EAAO and (**b**) EIO.

The as-prepared aniline tetramer-terminated EAAO and EIO were characterized by ATR-FTIR, as shown in Figure 2. In both of the ATR-FTIR spectra the characteristic band found at about 3259 cm^−1^ was attributed to the N-H stretching modes. Moreover, characteristic bands at 1580 cm^−1^ and 1506 cm^−1^ were attributed to C=C of quinoid rings and benzenoid rings, respectively [42,43]. The ATR-FTIR spectrum of EAAO was shown in Figure 2a, the characteristic band at 1653 cm^−1^ was attributed to C=O of CONH. After thermal imidization, the characteristic bands of the carboxyl groups vanished in the EIO spectrum, as shown in Figure 2b. Meanwhile, new characteristic absorption bands appeared at 1774 cm^−1^ and 1715 cm^−1^ of the EIO spectrum, which may be associated with asymmetric and symmetric carbonyl stretching. Moreover, the characteristic band found at 1378 cm^−1^ and 746 cm^−1^ was designated as the C-N stretching and deformation of the imide groups, respectively [44,45]. The above information indicates that the EAAO had been effectively converted into the corresponding EIO through the thermal imidization process.

The solubility of the synthesized electroactive amic acid oligomer in some common organic solvents is summarized in Table 1. The EAAO exhibited excellent solubility in many polar solvents such as NMP, DMF, DMAc, DMSO and THF. The high solubility of EAAO could be attributed to the following reasons: First, the flexible ether linkage (-O-) of IDPA, which reduced the rotation energy barrier of the molecule chain and secondly, the polar functional groups (carboxylic acid and amide) of EAAO, which enhanced the interaction between EAAO and the solvent [46]. The high solubility of the EAAO provides a distinct advantage in processing.

### 3.3. Chemical Oxidation of AT and EAAO

**Figure 3:**
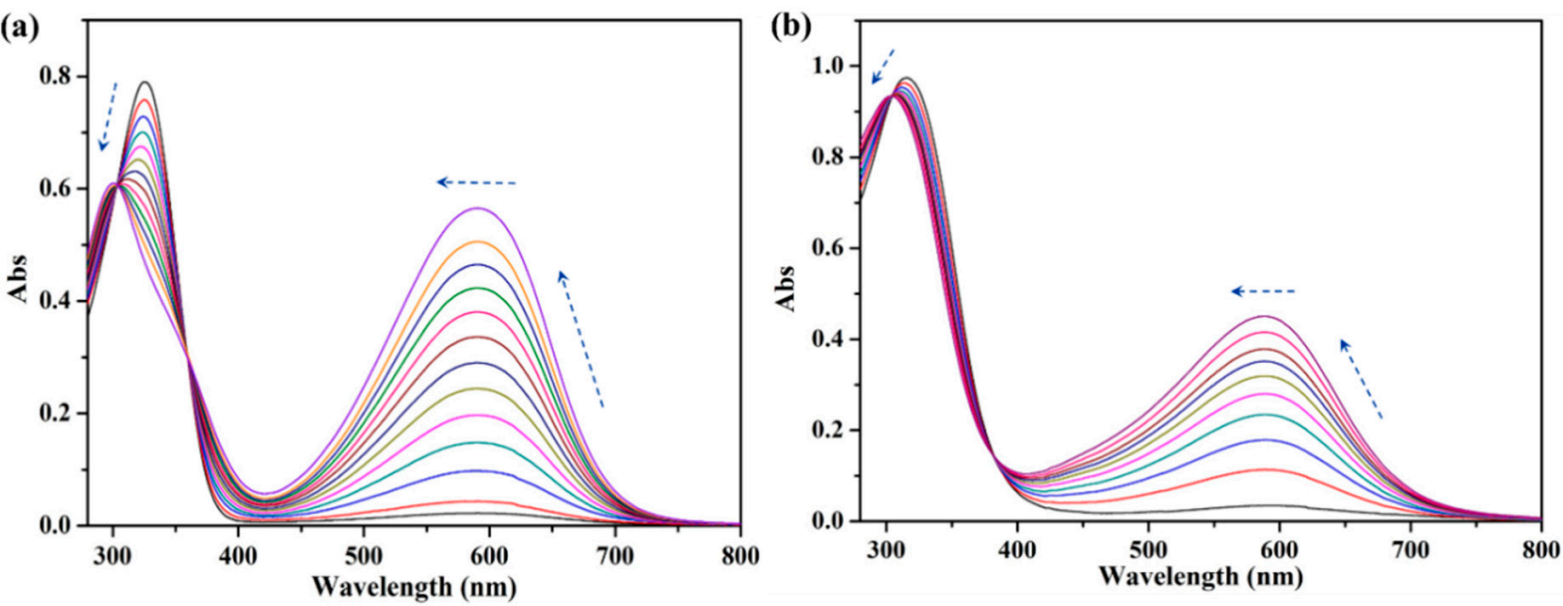
UV-Vis spectra monitoring chemical of (**a**) AT and (**b**) EAAO.

The obtained LB state of AT and EAAO were dissolved in NMP and ethanol, respectively. Subsequently, a trace amount of (NH_4_)_2_S_2_O_8_ was added to them. The clear solution gradually changed dark blue and then purple upon oxidization. This process was continuously monitored by UV-Vis spectra, as shown in Figure 3. Initially, in each of the UV-Vis spectra, only one absorption band (AT at 326 nm, EAAO at 316 nm) was observed which is associated with the π-π* transition of the conjugated ring system [47]. When it is slowly oxidized, the absorption undergoes a blue shift (AT from 326 nm to 300 nm, EAAO from 316 nm to 306 nm), with decreasing intensity. At the same time, a new absorption appeared (AT at 592 nm, EAAO at 590 nm) which was designated to the exciton-type transition between the HOMO of the benzoid ring and the LUMO orbital of the quinoid ring [48,49]. As the intensity reached its maximum, the second absorption underwent a blue shift (AT from 592 to 590 nm, EAAO from 590 to 580 nm) [45].

### 3.4. Electroactivity of AT, EAAO and EIO Coatings

**Figure 4:**
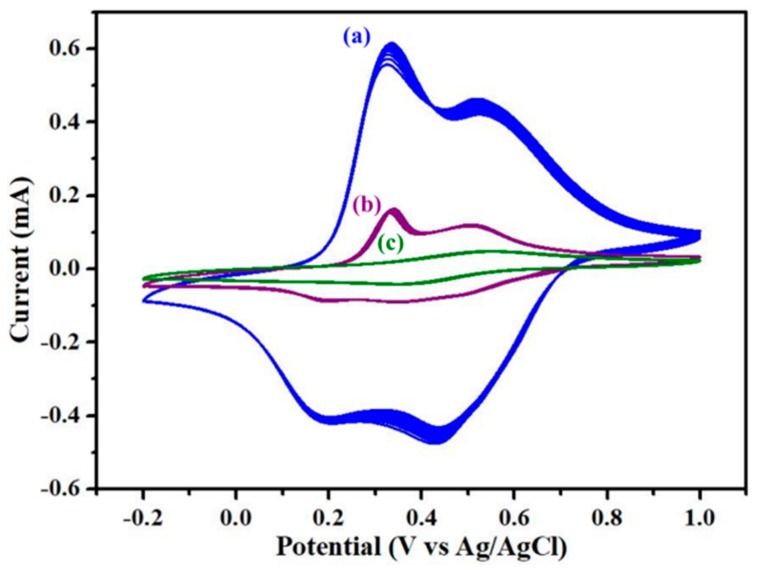
Cyclic voltammetry of (**a**) AT, (**b**) EAAO and (**c**) EIO were measured 10 cycles in aqueous H_2_SO_4_ (1.0 M) with scan rate of 50 mV s^−1^.

**Scheme 2:**
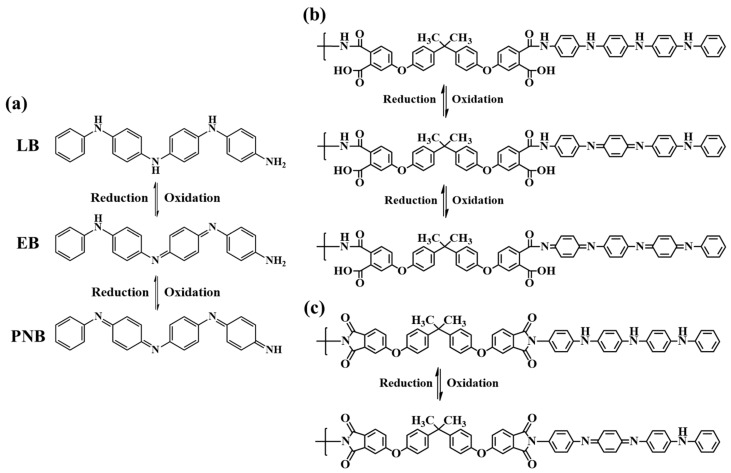
Molecular structures of (**a**) AT, (**b**) EAAO and (**c**) EIO at various oxidation states.

The cyclic voltammetry of AT, EAAO, and EIO using Ag/AgCl as the reference electrode was performed at a scan range of −0.2 to 1.0 V and a scan rate of 50 mV s^−1^ in 1.0 M H_2_SO_4_. As shown in Figure 4a, the cyclic voltammetry curve of the AT shows two pairs of redox peaks at 320 mV/200 mV and 520 mV/420 mV, which correspond to the transitions from the leucoemeraldine base (LB) to the emeraldine base (EB) and the emeraldine base to the pernigraniline base (PNB), respectively (Scheme 2a) [50]. Moreover, the CV curve of the EAAO shows two pairs of redox peaks at 330 mV/200 mV and 500 mV/350 mV, respectively, as shown in Figure 4b. In contrast, the CV results obtained from the EIO were different from those obtained from the EAAO. Only one pair of redox peaks at 360 mV/540 mV could be observed, as shown in Figure 4c. The differences in the EAAO and the EIO systems could be attributed to their molecular structures (Scheme 2b,c). In the case of the EIO system, the secondary amines at the EAAO segment were condensed during the thermal imidization with carboxylic acid to form an imine, which would not allow for the formation of quinoid rings. Thus, the EIO existed only in one oxidation state [51], as shown in Scheme 2.

### 3.5. Potentiodynamic Measurements

**Figure 5:**
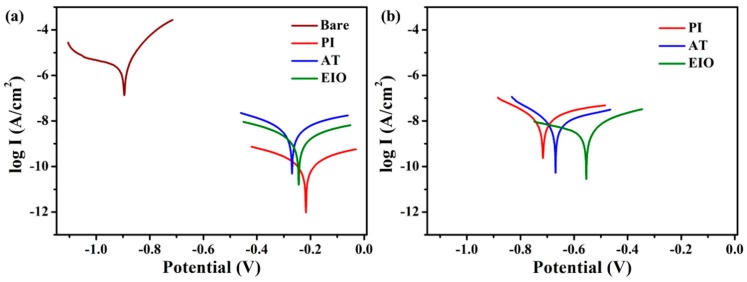
Tafel plots for the PI, AT and EIO coatings measured in 3.5 wt.% NaCl solution for (**a**) 1 day and (**b**) 30 days.

The polarization curves, electrochemical parameters of bare CRS, PI, AT, and EIO coatings, were exhibited in Figure 5. All the corrosion parameters including corrosion potential (*E_corr_*), corrosion current density (*I_corr_*), polarization resistance (*R_p_*), corrosion rate (*CR*), anodic Tafel slope (*b_a_*) and cathodic Tafel slope (*b_c_*) are summarized in Table 2. Generally, a higher value of *E_corr_* and *R_p_*, as well as lower values of *I_corr_* and *CR*, implied better corrosion protection [34,44,52,53]. Figure 5a shows that bare CRS had the lowest *E_corr_* (−0.89 V) and the highest *I_corr_* (2.52 × 10^−6^ A cm^−2^). By contrast, these values of the PI, AT and EIO coatings had their anti-corrosion performance improved. The values of *E_corr_* were increased to −0.21 V, −0.27 V and −0.24 V, respectively. Meanwhile, the values of *I_corr_* were decreased to 1.85 × 10^−10^ A cm^−2^, 4.30 × 10^−9^ A cm^−2^, and 2.86 × 10^−9^ A cm^−2^, respectively.

To further study the long-term anti-corrosion protection performance of the PI, AT and EIO coatings they were immersed in a 3.5 wt.% NaCl solution for 30 days. The *E_corr_* and *I_corr_* of the PI coating were found to be −0.71 V and 8.64 × 10^−9^ A cm^−2^, respectively. Furthermore, both the AT and EIO coatings exhibited better anti-corrosion performance than the PI, as shown in Figure 5b. The *E_corr_* of the AT and EIO coatings were −0.67 V and −0.55 V, respectively, while the *I_corr_* values were 7.85 × 10^−9^ A cm^−2^ and 3.32 × 10^−9^ A cm^−2^. The detailed values of corrosion parameters are summarized in Table 3. The results show that the EIO coating has the highest *E_corr_* and lowest *I_corr_*, thus it can be concluded that it demonstrated the strongest anti-corrosion performance.

### 3.6. Electrochemical Impedance Spectroscopy (EIS) Measurements

**Figure 6:**
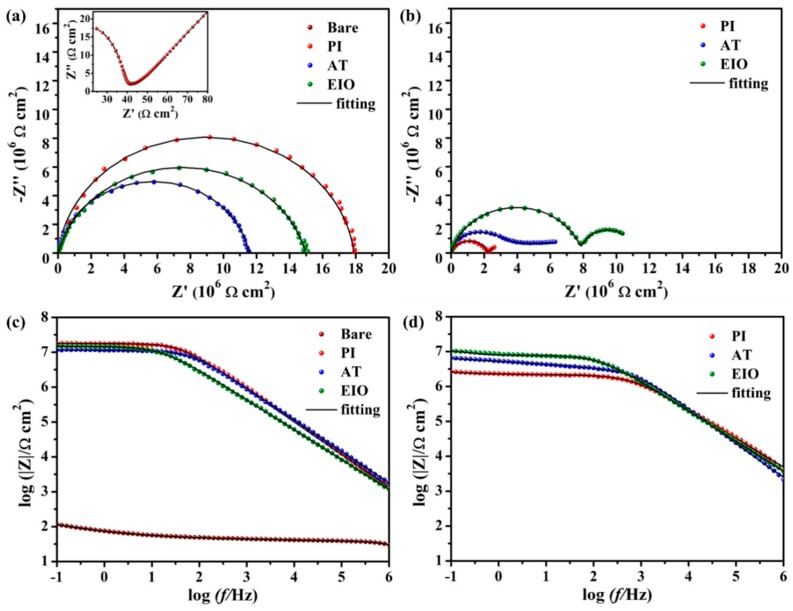
The PI, AT, and EIO coatings measured in 3.5 wt.% NaCl solution for Nyquist plots for (**a**) 1 day, (**b**) 30 days and Bode plots (**c**) 1 day and (**d**) 30 days. The inset shows the Nyquist plots for neat CRS.

**Figure 7:**
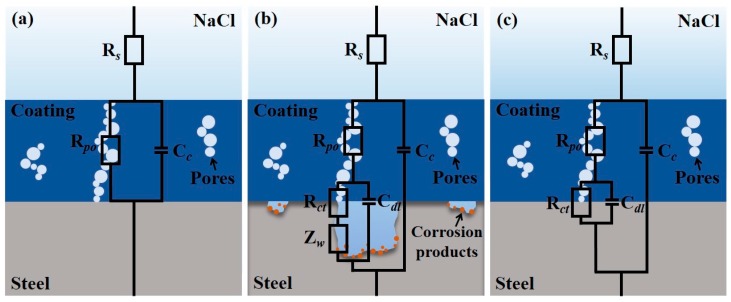
The equivalent electric circuits of (**a**) all of coatings immersion 1 day; (**b**) PI, (**c**) AT and EIO immersion 30 days.

To further investigate the corrosion of the CRS electrode coated by PI, AT and EIO, the EIS measurements were conducted in 3.5 wt.% NaCl solution. The EIS results are presented in Figure 6a. The equivalent electric circuits were shown in Figure 7. *R_s_* is the solution resistance between the working electrode and the reference electrode, *R_po_* and *C_c_* correspond to the coating pore resistance and coating capacitance, *R_ct_* and *C_dl_* represent the charge transfer resistance and double-layer capacitance, and *Z_w_* is the Warburg resistance [46]. The Nyquist and Bode plots of the neat CRS are shown as comparison studies. As shown in Figure 6a, at the initial immersion stage, all of the samples were semi-circular, indicating the CRS had increased anti-corrosion attributes [54,55,56,57]. The EIS data were fitted with the equivalent circuit in Figure 7a.

After long-term immersion, the Nyquist plots of the PI exhibited shrinking capacitive arcs followed by a tiny diffusion tail, indicating that the metal surface corrosion was caused primarily by a charge reaction [58,59,60]. Moreover, the electrochemical impedance spectra of AT and EIO show two time constants and two capacitive arcs, as shown in Figure 6b. The high-frequency capacitive arc reflected the properties of the coating, while the low-frequency capacitive loop reflected the corrosion of the metal beneath the coating [61]. The larger radius of the semicircle signified that the coating has a high impedance during the water seepage stage due to the passive metal oxide layers formed by the segments of aniline tetramer, hindering the penetration of water and oxygen and preventing further corrosion of the CRS substrate. The measured results of the EIS were fitted with the equivalent circuit in Figure 7b,c.

The Bode plots of the PI, AT, and EIO were shown in Figure 6c,d. In general, the lowest frequency region of the impedance modulus could be used to roughly estimate the coating resistance [62,63]. At the initial immersion stage, the |Z|_0.1 Hz_ value for bare, PI, AT and EIO were 1.14 × 10^2^, 1.79 × 10^7^, 1.15 × 10^7^ and 1.49 × 10^7^ ohm·cm^2^, respectively. After 30 days of immersion, the |Z|_0.1 Hz_ value of the PI, AT and EIO were dropped to 2.60 × 10^6^, 6.38 × 10^6^ and 1.05 × 10^7^ ohm·cm^2^, respectively. Both the AT and EIO coatings show better anti-corrosion effect than the PI during the long-term immersion period. The reason was attributed to the existing aniline tetramer units in the coatings, which can passivate the metal surface to form a protective iron oxide film. The results of the EIS were consistent with previous Tafel plot studies.

**Figure 8:**
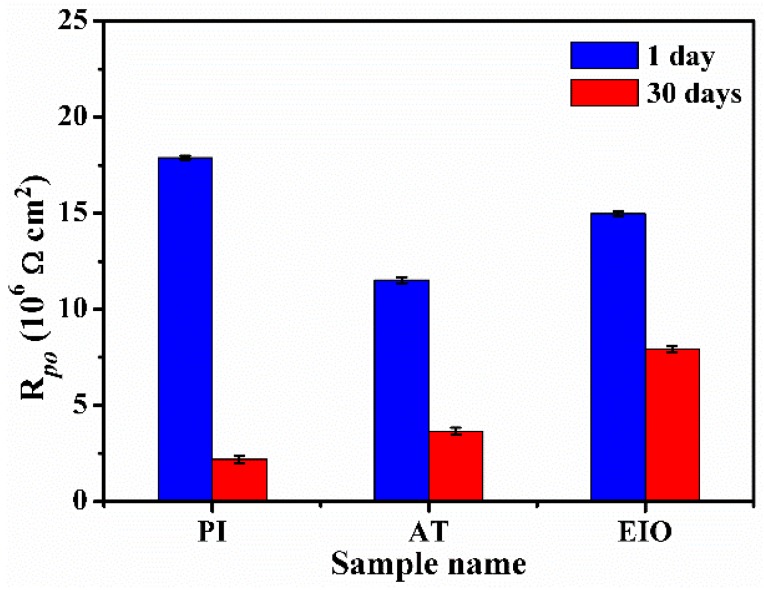
Coating pores resistance values of the PI, AT, and EIO coatings immersed in 3.5 wt.% NaCl solution for 1 day and 30 days.

Figure 8 shows the initial *R_po_* of all the coatings before and after the 30 days immersion period, after which the *R_po_* of all the samples had decreased due to defects in the coatings. The AT (3.66 × 10^6^ ohm·cm^2^) and EIO (7.93 × 10^6^ ohm·cm^2^) coatings show higher pore resistance than the PI (2.18 × 10^6^ ohm·cm^2^) due to the formation of passive metal oxide layers. Furthermore, the *R_po_* of the EIO was the highest among all the coatings, indicating that it offered the best protection for the CRS. This may be the result of the EIO coating not only forming passive metal oxide layers but also providing the charge transfer complex (CTC), a property that has been the subject of many studies [64]. CTC was formed between dianhydride and diamine groups in polyimide systems and is largely responsible for some of the valuable properties of polyimides. The increased interchain attractive forces resulting from such interactions are proposed to increase the rigidity of the chain, which increases the density of the EIO coating and grants it stronger anti-corrosion properties.

### 3.7. Corrosion Products and Corrosion Mechanism Analysis

**Figure 9:**
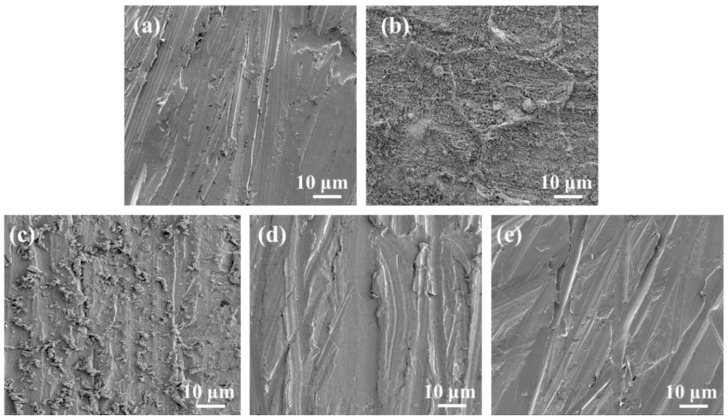
SEM images for (**a**) ground CRS, and the surface of CRS coated with the (**b**) bare, (**c**) PI, (**d**) AT and (**e**) EIO immersed in 3.5 wt.% NaCl solution duration for 30 days.

In further studies the SEM and XPS were used to confirm the formation of passive metal oxide layers (Fe_2_O_3_) upon the surface of the CRS. For comparison, the SEM image of a ground CRS surface is shown in Figure 9a. Figure 9b shows the ground CRS surface after the 30 days immersion period in a 3.5 wt.% NaCl solution, where dense, irregular projections and particles can be observed. Moreover, Figure 9c–d show the surface of the CRS where PI, AT, and EIO coatings were removed after a saline immersion treatment. It is observed from Figure 9c that sparse granular substances covered the surface of the CRS. This suggests that the coating is unable to completely suppress the penetration of the aggressive corrosive medium (chlorine and dissolved oxygen ions) into the underlying substrate during long-term immersion treatment. On the other hand, the AT and EIO coatings were relatively cleaner, as shown in Figure 9d,e. This indicates that a passive metal oxide layers formed on the CRS surface.

**Figure 10:**
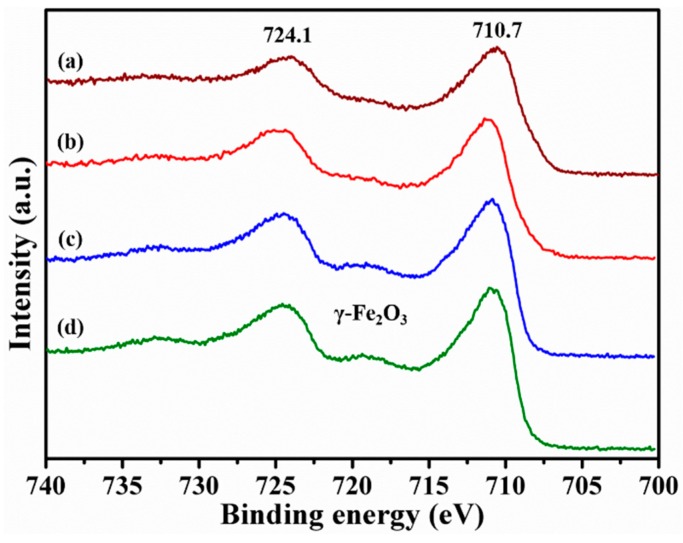
XPS Fe 2p core level spectra of (**a**) ground CRS, (**b**) PI, (**c**) AT, and (**d**) EIO.

**Figure 11:**
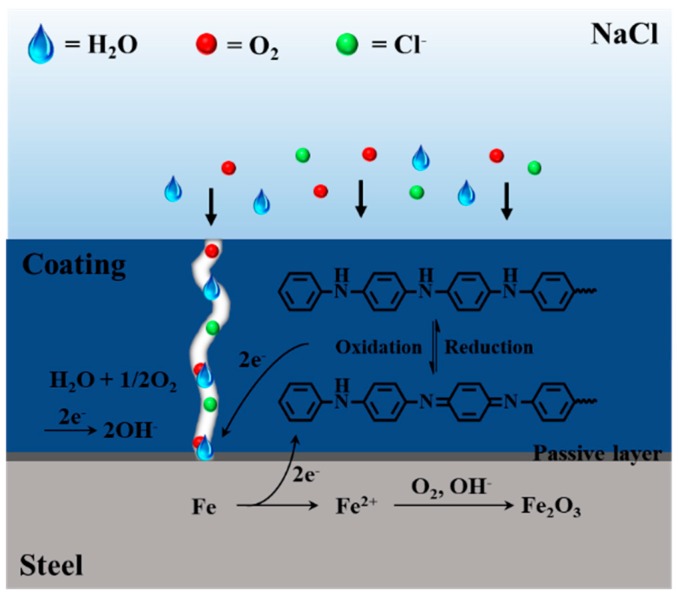
Schematic diagrams of the mechanism of CRS passivation by EIO coatings.

The chemical composition of the passivating oxide layers was determined by XPS. The binding energy vs. intensity plots for iron oxide layers are shown in Figure 10. For comparison, the XPS plots of the ground CRS surface and CRS surface beneath PI are shown in Figure 10a,b. As shown in Figure 10c,d, the CRS surface coated with the AT and EIO all exhibited two binding energy peaks of Fe 2p_3/2_ and Fe 2p_1/2_ of Fe_2_O_3_ at 710.7 eV and 724.1 eV, respectively. Furthermore, a distinctive shake-up satellite at around 719.1 eV indicates that the passivation oxide layer consists mainly of Fe_2_O_3_ [65,66]. The corrosion process in neutral solutions is presented in the following equations [67,68] and schematic diagrams (Figure 11):

anodic reaction:

Fe → Fe^2+^ + 2e^−^ (3)

O_2_ reduction takes place at the cathode:

1/2O_2_ + 2e^−^ + H_2_O → 2OH^−^ (4)

chemical process:

2OH^−^ + Fe^2+^ → Fe(OH)_2_ → Fe(OH)_3_ → Fe_2_O_3_ (5)

## 4. Conclusions

In this study, the aniline tetramer capped electroactive imide oligomer (EIO) was successfully synthesized and applied as an anti-corrosion coating for a cold-rolled steel electrode. The precursor of the EIO, electroactive amic acid oligomer (EAAO), is limited due to its high solubility in common organic solvents. The anti-corrosive properties of the EIO coating were evaluated by Tafel curves and EIS. Further investigation of the oxidation products underneath the coating using SEM and XPS concluded that the EIO coating facilitated the formation of passive metal oxide layers. This study offers a novel method to prepare the soluble electroactive oligomer, which might be useful when applied to protecting metals from corrosion.

## Figures and Tables

**Table 1:** Solubility of EAAO in common organic solvents ^a^.

	NMP	DMF	DMAc	DMSO	THF	CHCl_3_	CH_2_Cl_2_	Acetone	Ethanol
EAAO	+ +	+ +	+ +	+ +	+ +	+	+	+	+

^a^ The solubility of the EAAO was determined with 5% solid content at 25 °C. ++ = soluble, + = partially soluble.

**Table 2:** Electrochemical parameters derived from Tafel plots of the coatings in the saline solution after one-day immersion.

Sample	Electrochemical Measurements						
	E_corr_ (V)	I_corr_ (A cm^−2^)	R_P_ (Ω cm^2^)	*CR* (mm/year)	b_a_	b_c_	Thickness (μm)
**Bare**	−0.89	2.52 × 10^−6^	8.69	2.90 × 10^−2^	0.060	0.309	-
**PI**	−0.21	1.85 × 10^−10^	3.55 × 10^8^	2.17 × 10^−6^	0.305	0.300	22 ± 1
**AT**	−0.27	4.30 × 10^−9^	1.22 × 10^7^	5.03 × 10^−5^	0.260	0.226	21 ± 1
**EIO**	−0.24	2.86 × 10^−9^	2.88 × 10^7^	3.35 × 10^−5^	0.421	0.344	22 ± 1

**Table 3:** Electrochemical parameters derived from Tafel plots of the coatings in the saline solution after 30 days immersion.

Sample	Electrochemical Measurements						
	E_corr_ (V)	I_corr_ (A cm^−2^)	R_P_ (Ω cm^2^)	*CR* (mm/year)	b_a_	b_c_	Thickness (μm)
**PI**	−0.71	8.64 × 10^−9^	3.66 × 10^6^	1.01 × 10^−4^	0.162	0.132	22 ± 1
**AT**	−0.67	7.85 × 10^−9^	5.33 × 10^6^	9.19 × 10^−5^	0.269	0.150	21 ± 1
**EIO**	−0.55	3.32 × 10^−9^	1.33 × 10^7^	3.89 × 10^−5^	0.138	0.385	22 ± 1
